# Extending Transfer Entropy Improves Identification of Effective Connectivity in a Spiking Cortical Network Model

**DOI:** 10.1371/journal.pone.0027431

**Published:** 2011-11-15

**Authors:** Shinya Ito, Michael E. Hansen, Randy Heiland, Andrew Lumsdaine, Alan M. Litke, John M. Beggs

**Affiliations:** 1 Department of Physics, Indiana University, Bloomington, Indiana, United States of America; 2 School of Informatics, Computer Science Group, Indiana University, Bloomington, Indiana, United States of America; 3 Santa Cruz Institute for Particle Physics, University of California Santa Cruz, Santa Cruz, California, United States of America; 4 Biocomplexity Institute, Indiana University, Bloomington, Indiana, United States of America; University of Michigan, United States of America

## Abstract

Transfer entropy (TE) is an information-theoretic measure which has received recent attention in neuroscience for its potential to identify effective connectivity between neurons. Calculating TE for large ensembles of spiking neurons is computationally intensive, and has caused most investigators to probe neural interactions at only a single time delay and at a message length of only a single time bin. This is problematic, as synaptic delays between cortical neurons, for example, range from one to tens of milliseconds. In addition, neurons produce bursts of spikes spanning multiple time bins. To address these issues, here we introduce a free software package that allows TE to be measured at multiple delays and message lengths. To assess performance, we applied these extensions of TE to a spiking cortical network model (Izhikevich, 2006) with known connectivity and a range of synaptic delays. For comparison, we also investigated single-delay TE, at a message length of one bin (D1TE), and cross-correlation (CC) methods. We found that D1TE could identify 36% of true connections when evaluated at a false positive rate of 1%. For extended versions of TE, this dramatically improved to 73% of true connections. In addition, the connections correctly identified by extended versions of TE accounted for 85% of the total synaptic weight in the network. Cross correlation methods generally performed more poorly than extended TE, but were useful when data length was short. A computational performance analysis demonstrated that the algorithm for extended TE, when used on currently available desktop computers, could extract effective connectivity from 1 hr recordings containing 200 neurons in ∼5 min. We conclude that extending TE to multiple delays and message lengths improves its ability to assess effective connectivity between spiking neurons. These extensions to TE soon could become practical tools for experimentalists who record hundreds of spiking neurons.

## Introduction

To understand the functioning of a complex system, it is often useful to develop a map of interactions between the system's components. This “network science” approach has been applied to a wide variety of systems with great success [1]–[5].

Typically, interactions between components are classified as physical, functional, or effective [6], [7]. In neuroscience, a synapse or gap junction would constitute a physical connection between neurons; a correlation between spike trains of two neurons would constitute a functional connection; the ability to predict the firing of one neuron based on the firing of another neuron would constitute an effective connection.

Physical connections delimit the ways in which activity *could* flow within a circuit, whereas effective connections describe the ways in which activity *typically* flows. Note that effective connections can be a small subset of physical connections. Thus, knowledge of effective connectivity may provide insights into how information is typically distributed and recombined in neural circuits. An effective connectivity map would permit many powerful graph-theoretic tools to be applied [8]–[10], potentially allowing subtle differences in information processing between healthy and diseased networks to be identified. Now that it is possible to record activity from hundreds of closely spaced neurons at high temporal resolution for several hours at a time [11]–[14], it is extremely important to have an accurate and robust measure of effective connectivity between neurons.

In this regard, transfer entropy (TE) has recently received much attention in neuroscience [15]–[19]. It is an information-theoretic measure first introduced by Thomas Schreiber [20] to assess effective connectivity. As an information-theoretic measure, it has often been claimed that TE can be used to estimate “information flow” between neurons [15], [21]. In a recent paper that surveyed several different information theoretic methods [16], the authors concluded that TE performed better at identifying effective connectivity in complex systems than cross-correlation (CC), mutual information, and joint entropy. Another recent study compared TE to extended and nonlinear Granger causality, and to predictability improvement. These authors concluded that TE was superior in terms of stability and accuracy when applied to time series from linear and non-linear models with unidirectional and bidirectional coupling [22]. Thus, although there are many potential ways to assess interactions between neurons including Granger causality [23]–[26], the directed transfer function (DTF) [24], [27], generalized linear models [28], Bayesian models [29], data mining techniques [30], [31], partial directed coherence [32], [33], and many others [34]–[46], TE is gaining wide acceptance.

Despite the accuracy of TE, it has several limitations. First, most of the published work using TE has assessed interactions at only one time delay [15]–[18], but see [21], [47]. This is problematic because delays between an action potential and a post-synaptic potential typically range from one to twenty milliseconds in the mammalian cortex [48]–[50]. To identify such connections with single-delay TE (D1TE), one might try to use large time bins (∼20 ms), but this could sacrifice temporal resolution. This suggests a need to investigate TE with multiple delays. Second, all of the authors to our knowledge have only considered messages between neurons of a single bin length. While such measures would capture information sent between neurons in the form of a single spike, they would exclude messages spanning multiple time bins, like bursts. As bursts are widely hypothesized to be an important type of message sent between neurons [51], [52], it would be desirable to measure TE with message lengths beyond one bin. To resolve these problems, we here introduce an extension to TE that allows us to probe connectivity at multiple time delays (due, for example, to synaptic connections or axonal signal propagation), selecting the peak value of TE. We call this delayed TE. Furthermore, we also extend Delayed TE by considering message lengths up to 5 bins long at multiple delays. We call this higher-order TE (HOTE). We offer these extensions as freeware on our project website (http://code.google.com/p/transfer-entropy-toolbox/, Text S1).

To evaluate these extensions to TE (delayed TE, HOTE), we wanted to compare them to D1TE and CC. We selected CC because, in its time-lagged form, it has been widely used to assess effective connectivity between neurons [53], [54]. The CC does not produce a single number, but a set of numbers indicating the strength of a connection at various delays. It is thus necessary to develop a method for comparing CC curves. Interestingly, we found that there was not complete agreement in the literature on this topic. In particular, some research papers did not specify a method for normalizing the CC, even though this can substantially affect results. To alleviate this situation, we adopt a standard coincidence index (CI) [55]–[57]. The CI allows the peak region of a measurement to be selected when it is assessed at many delays. Accordingly, we applied the CI to delayed TE, HOTE and to CC.

As our eventual goal is to assess effective connectivity in experimental data, we applied our methods to a neural network model as a first step in validating our approach. Izhikevich's cortical network model [58] is widely used as it is computationally inexpensive and has many realistic features including cell types with different intrinsic firing patterns [59], different synaptic delays, and spike-timing dependent plasticity (STDP) [60]. In addition, this model is known to capture several emergent properties present in cortical circuits, including gamma oscillations [61] and repeating patterns of spike activity (polychronous groups) [58]. These emergent phenomena pose severe challenges for methods of assessing effective connectivity, as they frequently produce situations in which many neurons appear to be driving another neuron later in time. We applied our methods to Izhikevich networks containing 1000 neurons. Previous groups have validated their methods in simplified circuits containing relatively few model neurons [16], [43], on a few neurons embedded in a larger network [25], or in networks with only a single time delay [16], so the task posed here is challenging. By using a model with known connectivity, we were able to measure the true positive and false positive rates of each method, allowing for objective comparisons of performance.

## Materials and Methods

### Model network

The simulated network on which the connectivity algorithms were tested was based on Izhikevich's network model [58]. The program was copied directly from his article (and is reproduced in our supporting information, Code S1) and only slightly modified as we describe below. The reader should consult the source code for details. Here we give an overview of the four main aspects of the model: intrinsic neuron dynamics, synaptic inputs and plasticity, connectivity, and implementation.

#### Intrinsic neuron dynamics

The voltage of each simulated cortical neuron was described by the coupled differential equations:
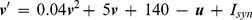
(1)

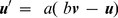
(2)

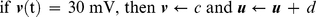
(3)where ***v*** was the neuron's voltage, ***v′*** was the time derivative of the voltage, ***u*** was a recovery variable, ***u′*** was the time derivative of the recovery variable, *I_syn_* was the total synaptic input received by the neuron (see below), and *a*, *b, c* and *d* were adjustable parameters that governed the firing behavior of the neuron. Here, the notation (***v*** ←*c*) indicates that the variable ***v*** will be assigned the value of *c* if the conditional statement is true. The units of time were milliseconds and the units of voltage were mV. Izhikevich found that by adjusting *a, b, c* and *d*, he could mimic a wide variety of intrinsic firing patterns found in cortical neurons, including those from regular spiking (RS), intrinsically bursting (IB) and fast spiking (FS) neurons[59], [62]. To simulate RS cells, we used (*a, b, c, d*)  =  (0.02, 0.2, −65, 8). To simulate FS cells, we used (*a, b, c, d*)  =  (0.1, 0.2, −65, 2). The model was populated with 800 RS cells and 200 FS cells only.

#### Synaptic inputs and plasticity

The total synaptic input arriving at a cell, *I_syn_*, represented thalamic input as well as input from other cortical neurons. Excitatory thalamic input was delivered at random times, simulated as a Poisson process with a mean firing rate of 1 Hz. Each thalamic input had a synaptic weight of 20 mV. Input from inhibitory cortical neurons had a weight of 5 mV; input from excitatory cortical neurons initially had synaptic weights of 6 mV. Although there was no single spike threshold in this model, it is clear that a single input by itself could not drive a neuron to spike. These excitatory weights were then evolved by spike-timing dependent plasticity (STDP) in the following manner: synaptic inputs that arrived shortly before a postsynaptic spike had the derivatives of their weights incremented, and synaptic inputs that arrived shortly after a postsynaptic spike had the derivatives of their weights decremented. This algorithm led to slow weight changes. The amount of increment or decrement was large when the presynaptic spike time was close to the postsynaptic spike time. It was less when the presynaptic spike time was far from the postsynaptic spike time. This decay in increment (or decrement) fell off exponentially with a time constant of 20 ms.

#### Connectivity

Each neuron had 100 delayed synaptic connections to other neurons. Connections from excitatory neurons had a uniform distribution of delays from 1 ms to 20 ms. Connections from inhibitory neurons had a fixed delay of 1 ms. Inhibitory neurons were only connected to excitatory neurons, while excitatory neurons could be connected to any neurons.

#### Implementation

**Figure 1:**
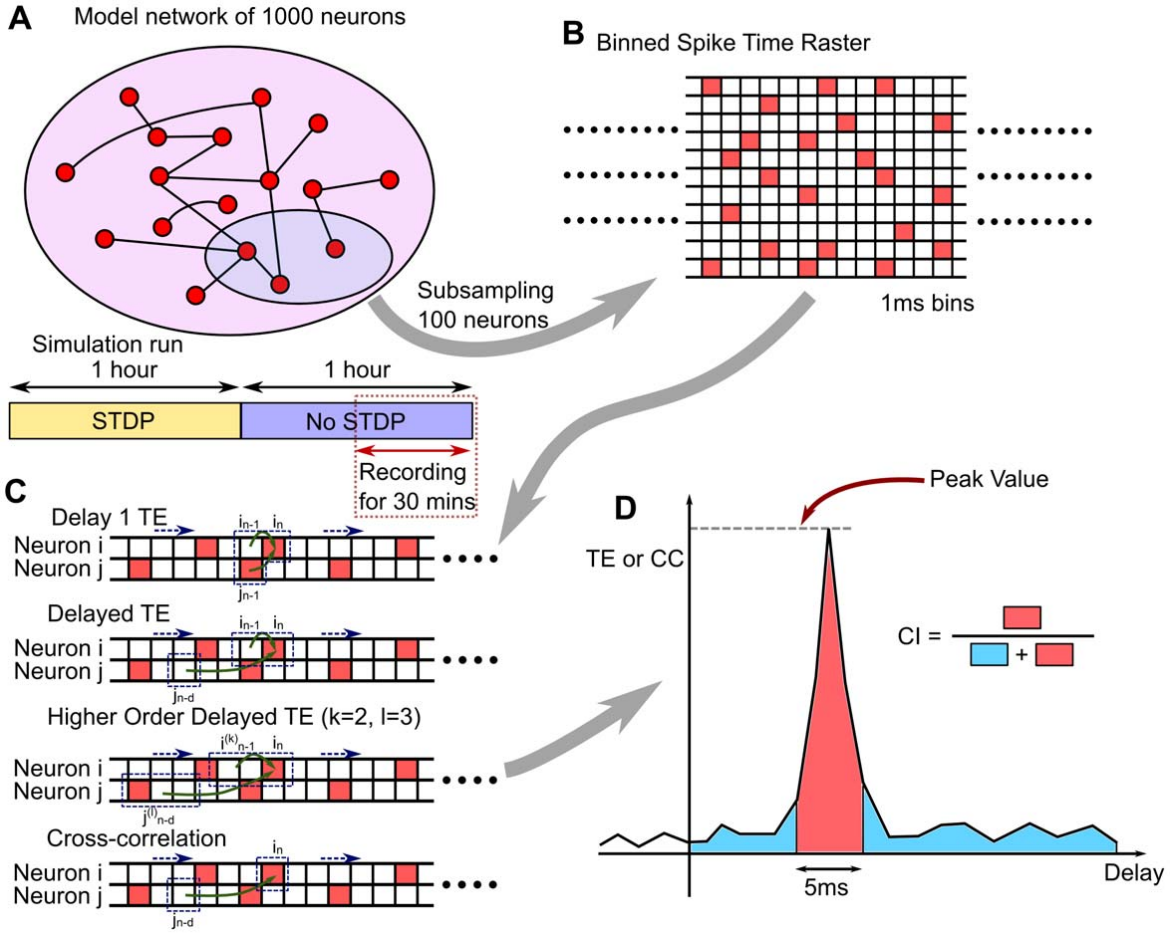
Overview of methods. A: 1000 neurons in an Izhikevich network are simulated with STDP active for 1 hour. STDP is terminated at 1 hour and simulation is run for another hour. The last 30 minutes of the simulation is recorded, and 100 neurons are randomly subsampled for the analysis. B: Spike Time raster is created from the spikes of simulated neurons. The spikes are binned with 1ms time bins to form binary time series. C: Extensions of TE. Keeping the spike time history of Neuron *i* the same, we shifted the relative timing of the spike of Neuron *j*. The same method can also be applied to higher order TE which looks at multiple bins for each neuron. CC looks at only one bin from each system, and does not use the information of Neuron *i*'s history. D: A schematic of delayed TE or CC (not from the data) illustrating Coincidence Index (CI) calculation. Peak value is the maximum of the measure, and CI is fraction of area of 5 ms window around the peak. If the distribution is peaked, CI tends to have a high value.

To approximate electrode array recordings that sub-sample from a population of cells, only 80 excitatory and 20 inhibitory neurons were randomly sampled from the entire 1000 neuron network for connectivity analysis (figure 1A). The simulation was run for 2 hrs of simulated time. STDP was enabled in the first hour, and was stopped in the second hour to fix the synaptic weights. Only the last 30 minutes of output were used to evaluate connectivity algorithms, so as to avoid model transients. The differential equations of the model were evaluated in 0.5 ms intervals while spike times were stored in 1 ms time bins (figure 1B). Each simulation was repeated with a different random number seed for a total of 8 different data sets for analysis. Because of synaptic plasticity, excitatory connections had various synaptic weights, ranging from 0 to 10 mV. Only synapses with weights greater than 1 mV were considered as potentially driving connections, and considered true connections in our performance analysis. As stated in the results section, many of the ignored connections are close to 0 mV, and the fraction of the ignored portion of synaptic weights was only 0.5% of the total.

### Definition of effective connectivity

Friston [6] described effective connectivity as “the influence one system exerts over another.” Thus, effective connections are directed from one neuron to another, as opposed to functional connections, which are undirected. In a similar manner, Norbert Wiener [34] sought to describe couplings between units in a complex system by the ability to predict time series. He thought that an effective connection existed from system A to system B when knowledge about the past of system A improved the ability to predict the future of system B, beyond the prediction based on the past of system B alone. Although there are many definitions of effective connectivity in the literature [6], [45], [63], we will here adopt Wiener's notion of predictability improvement, as this seems to capture the most common central idea.

### Delay-one transfer entropy (D1TE)

TE is a directed (asymmetric), measure of interaction that can be applied to two time series. In neuroscience terms, TE is positive if including information about neuron *J*'s spiking activity improves the prediction of neuron *I*'s activity beyond the prediction based on neuron *I*'s past alone. Thus, TE is also called an information flow measure [15], [21]. The original definition of TE [20] was given as
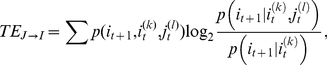
(4)where *i_t_* denoted the status of neuron *I* at time *t*, and could be either 1 or 0, indicating a spike or no spike, respectively; *j_t_* denoted the status of neuron *J* at time *t*; *i_t+_*_1_ denoted the status of neuron *I* at time *t+*1; *p* denoted the probability of having the status in the following parentheses; and the vertical bar in the parentheses denoted the conditional probability. The sum was over all possible combinations of *i_t+1_*, *i _t_^(k)^*, and *j_t_^(l)^*. The parameters *k* and *l* gave the order of TE, meaning the number of time bins in the past that were used to calculate the histories of systems *I* and *J*, respectively. We defined ‘Delay 1 TE’ (D1TE) by equation (4) when *k* = *l* = 1, so that only single time bins were considered. We used logarithms with base 2 so that our units would be bits.

The key advantage of TE over CC was the use of its own history. This played an important role when the behavior of the system depended on its own history, as in the case of refractory periods after spiking. The obvious weakness of D1TE was that it only assessed one time bin from the spike of one neuron. Any information with longer delay times was missed.

### Delayed transfer entropy (TE)

We wanted to extend D1TE to increase its temporal range while retaining its advantages. To continue to consider the system's own history, we kept a 1-bin delay for neuron *I*, assuming that neuron *I* depended mostly on its closest previous state (figure 1C). This assumption was justified for our application, as the mutual information between bins for a single spike train peaked at a delay of one in the model. To account for synaptic delays between neurons, the time bin of neuron *J* was allowed to shift from 1 to 30 bins.

Taking these modifications into account, we defined delayed transfer entropy (TE) in the following way:
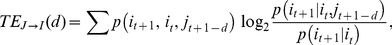
(5)where *d* was the time delay of interest, and other terms were as defined above for D1TE. We also used first-order parameters (*k* = *l* = 1) in this definition. Now that TE was defined as a function of time delay, it could be compared to CC, which was defined as a function of time differences. This allowed us to use methods that had been developed for CC, like the coincidence index (CI), on delayed TE.

### Higher-order transfer entropy (HOTE)

In most previous applications of TE [15], [16], [64], the order (*k*, *l*) was set to 1 for two reasons: to make computation faster, and to ensure adequate sampling. For binary spike trains, the number of patterns considered in first-order TE was 2^3^ = 8. As the order was increased, the number of patterns considered increased exponentially (2^(*k*+*l*+1)^), making higher-order TE computationally costly. In higher-order TE, the number of appearances of each pattern also decreased for a fixed data size. Nevertheless, we explored the potential utility of higher-order (HOTE) by examining message lengths up to 5 bins in both neurons *I* and *J*, and at all delays from 1 to 30 bins. More specifically, we explored HOTE for all 750 possible combinations of *k* =  (1, 2, 3…5), *l* =  (1, 2, 3…5), *d* =  (1, 2, 3…30). The equation for HOTE, incorporating these multiple message lengths and delays, was given by
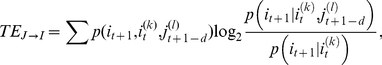
(6)where all symbols were as defined previously.

### TE Algorithm

We were able to search many combinations of delays and message lengths for extended TE because we used a relatively fast algorithm. The details of this algorithm, along with a software package and sample data set for MATLAB, are included on our project website (http://code.google.com/p/transfer-entropy-toolbox/, Text S1). Very briefly, the main feature of the algorithm that allowed it to calculate TE rapidly was that it only counted configurations for neurons *I* and *J* where at least one spike existed. The number of completely unoccupied bins was inferred from the number of occupied bins and the length of the data set. This short-cut prevented unnecessary counting of empty bins, and lead to substantial savings, as spike train data sets were typically very sparse, with many more unoccupied time bins than occupied time bins.

### Cross correlation (NCC, NCCH)

Even though CC appears extensively in the literature, remarkably there has been no standard definition or usage. We used two of the most popular definitions of CC for this study.

The first is what we call the Normalized Cross-Correlation (NCC) which was defined at time τ as:
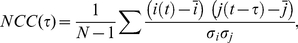
(7)where *i*(*t*), *j*(*t*) were the binary (1 or 0) states of neurons *I* and *J*, respectively, at time *t*. Here σ was the standard deviation and the overbar denoted the mean. *N* was the total number of time bins. This definition originated in the definition of the correlation index shifted by time. A similar definition was used in [65].

Another definition used in the literature is the Normalized Cross-Correlation Histogram (NCCH). Normalization of this measure has been somewhat controversial. We adopted the method that was used in [66]–[69], which used the geometric mean of the total number of firings of both neurons in a pair. The definition that we used for the NCCH at time τ was:
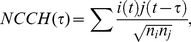
(8)where *n_i_*, *n_j_* were the total number of spikes from neurons *I* and *J*, respectively. For sparse spike trains, NCC and NCCH become similar to each other because NCC becomes NCCH in the limit as the averages of *i* and *j* approach 0 (sparse spike limit). However, if *i* and *j* become large, this can lead to difficulties in normalization.

### Connectivity identification (Coincidence Index, Peak)

It is important to note that Delayed TE and CC did not, by themselves, give a single number for each pairwise measurement, but rather provided many measurements for each time delay. In order to compare measures across different pairs of neurons, we needed to define a number that characterized the ‘strength’ of a connection, given these functions. One straightforward method was to take the maximum value of the function. We used this “peak” approach as one of the measures. We denoted a peak measure by appending a Pk suffix. For example: TEPk indicated transfer entropy measured at the peak value.

We also explored another well-known measure called the Coincidence Index (CI) [55]–[57], which was defined as: 
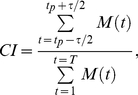
(9)where *M*(t) was the delay-dependent measure of interest, such as Delayed TE or CC, and *t_p_* was the time at which the measure peaked, τ was the coincidence window size, *T* was the entire window size of the measure (figure 1D). In this study we set *T* = 30 ms and τ = 5 ms. The choice of τ was based on the fact that the spike timing of postsynaptic neurons displayed a jitter of approximately ±2 ms. Results varied gradually with different values of τ, and we found 5 ms to be best among 1, 3, 5, 7 ms. The CI could be thought of as estimating the ratio: signal/(signal+background), where the area under the peak would be mainly the “signal”. The window size *T* was a free parameter. For some analyses, peak values (Pk) were also chosen from a selection of delays ranging from 1 to 30 ms. We denoted a coincidence index measure by appending a CI suffix. For example: TECI indicated transfer entropy measured by the coincidence index method.

NCC could take on either positive or negative values, which caused problems in defining CI (the denominator could be near zero). We took the absolute value of the NCC to resolve this. Taking the absolute value also made it possible to see negative correlations that were caused by inhibitory connections.

We used these two methods to express the strengths of connections between neuron pairs. These values then were sorted in descending order, and different threshold values were applied so that the putative connections identified by our analyses could be compared with the connections known to exist in the network model.

### Glossary

As there are many acronyms used to describe connectivity measures, here we give a brief summary of the terms:

D1TE: Transfer entropy measured at a delay of one time bin, with order one (message lengths limited to one time bin). This is the form of transfer entropy typically used in most neuroscience studies.

TE: Delayed transfer entropy, measured at delays from 1 to 30 time bins, with order one (message lengths limited to one time bin). This is one way of extending transfer entropy that we and others [21] have recently adopted.

HOTE: Higher-order transfer entropy, measured at delays from 1 to 30 time bins, with order ranging from 1 to 5 (message lengths for both the presynaptic and postsynaptic neuron allowed to range up to five time bins). This is a more general extension of transfer entropy, first introduced in this paper.

NCC: Normalized cross correlation, which measures correlation between the deviations of two variables from their respective means.

NCCH: Normalized cross correlation histogram, which measures correlation between the values of two variables.

-CI: A suffix that could be appended to any of the above measures, it indicates that the strength of the connection is quantified by the coincidence index, a ratio of the area under the peak of the curve to the total area under the curve.

-Pk: A suffix that could be appended to any of the above measures, it indicates that the strength of the connection is quantified by the peak, or maximum height, of the curve.

### Receiver Operating Characteristic (ROC)

All of the algorithms described above were designed to perform an identification task, where “true positive” (TP) cases were to be distinguished from “true negative” (TN) cases. In the present application, a TP case would occur when an algorithm identified a connection between two neurons and there was in fact a synaptic connection between the two neurons in the model. A TN case would occur when an algorithm identified no connection between two neurons and there was in fact no synaptic connection between the neurons in the model. The receiver operating characteristic (ROC) curve is often used to evaluate the performance of algorithms in situations like this [16], [70], [71]. The ROC provides an objective, non-parametric way to quantify the performance that would be obtained if an ideal observer had access to a particular algorithm. To continue to define relevant terms, a false positive (FP) case would occur when an algorithm identified a connection when there was in fact no synaptic connection in the model (this is also called a type I error); a false negative case (FN) would occur when an algorithm identified no connection when there was in fact a synaptic connection in the model (this is also called a type II error). We will use TP, TN, FP, and FN to represent the number of true positive, true negative, false positive, and false negative cases, respectively.

The true positive rate (TPR) and the false positive rate (FPR) are given by the following equations:
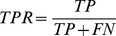
(10)

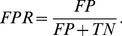
(11)


An ROC curve was obtained by plotting the TPR against the FPR as a discrimination threshold was gradually lowered (i.e., as connections with lower and lower values of Pk or CI were included).

In this framework, TP+FN was the total number of true connected pairs, and FP+TN was the total number of true unconnected pairs. It is important to note that in a sparsely connected network, as one would expect in cortical areas [72], the number of connected pairs was likely to be much smaller than the number of unconnected pairs. As we describe below, in our data the denominator of the TPR was likely to be much smaller than the denominator of the FPR (more than 10-fold). We therefore needed to be careful in interpreting the resulting curve. In particular, even though the TPR might have been larger than the FPR, the number of TP cases might have been smaller than the number of FP cases, which would have caused more false connections to be identified than true connections. To prevent this situation, we evaluated the performance of all algorithms at a FPR = 0.01. This ensured that the preponderance of connections was correctly identified, as described in the results below.

To further characterize the performance of these effective connectivity measures, we also evaluated the purity (sometimes called the “positive predictive value”, PPV) [73] of the connections found. Purity was given by:
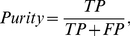
(12)where TP and FP were as defined above. Note that purity differs from the true positive ratio, TPR, in that the denominator is the total number of ‘positive predictions’ rather than the total number of ‘true’ connections. This approach has been adopted in other signal detection studies [73] and is especially useful when the number of true signals and false signals is largely different. High purity ensures that most of the positive predictions are correct.

## Results

### Characteristics of the simulated data

After the cortical network simulation stabilized, the mean firing rate of the excitatory neurons was 3.8±0.8 Hz (mean ± s.d.) and of the inhibitory neurons was 30.3±3.6 Hz. These average firing rates were stable over many minutes of simulated activity, but gamma-range (30 Hz) network-wide rhythmic activity did occur as reported in [58], leading to very short time scale fluctuations in firing rate. Under the influence of STDP, excitatory synaptic weights tended to settle near either their maximum value (∼10 mV) or their minimum value (∼0 mV). On average, 34.4 ± 1.4% of excitatory connections were below 1 mV. As stated in the methods, we excluded these connections from ROC analysis and estimated network information flow only for stronger connections. The synaptic weight of such excluded connections was only 0.5% of the total synaptic weight. This very small fraction of synaptic weight suggested that such connections could be excluded in an estimate of network effective connectivity. Connectivity density, which is the number of the connected pairs divided by the total number of pairs, of the network dropped from 10% (100 connections from each neuron) to 7.3% after the exclusion of these weak connections.

### Comparison of all the measures on 30 min data

**Figure 2:**
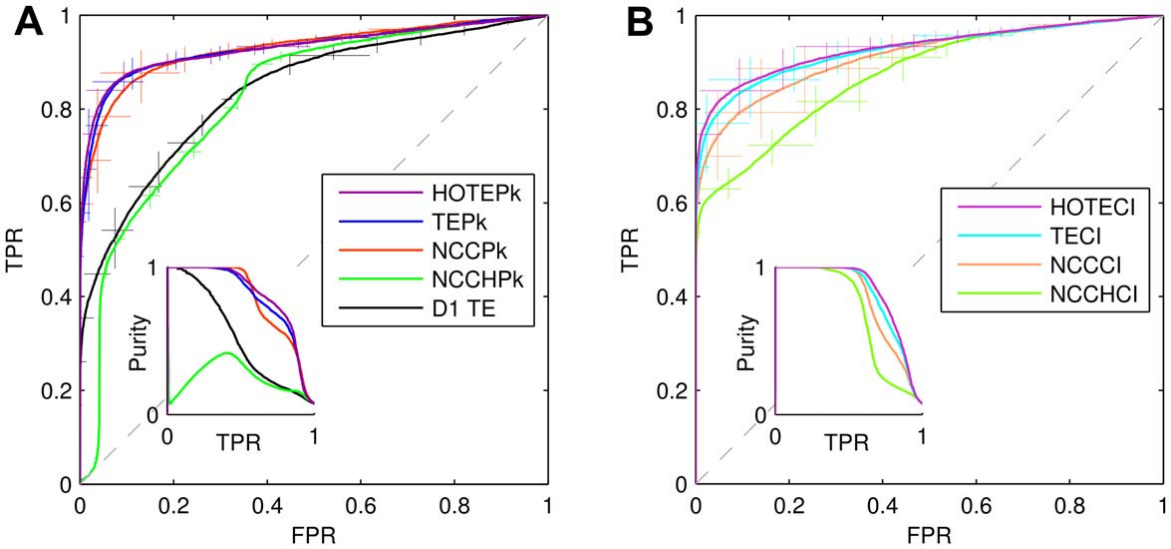
Comparison of effective connectivity measures. A: (left panel) peak values for all measures compared. B: (right panel) coincidence index values of all measures compared. The true positive rate (TPR) of detection is plotted against the false positive rate (FPR). These Receiver Operating Characteristic (ROC) curves were drawn by applying each measure to 30 minutes of data. Error bars were calculated at several positions based on 8 repetitions of the simulation. Vertical errors show standard deviation of TPR with fixed FPR; horizontal errors are that of FPR with fixed TPR. Inset shows purity plotted against TPR. In panel A, note that transfer entropy at delay of one time bin (D1TE) is clearly inferior to higher order transfer entropy peak (HOTEPk) and transfer entropy peak (TEPk).

Using 30 minutes of data from this model, we next investigated the performance of each measure (figure 2). We will first consider connections indicated by peak values (Pk); then we will consider those given by coincidence index values (CI). In figure 2A, we see that at almost all false positive rates, three measures (HOTEPk, Delayed TEPk and NCCPk) clearly outperform two others (D1TE and NCCHPk). This is also true when the false positive rate is 0.01, our chosen level for comparison. Of the peak measures, the performances at FPR = 0.01 of HOTEPk, TEPk, and NCCPk are equivalent within the errors, as can be seen from Table 1. The inset of figure 2 A shows that purity was near 1 for values of TPR that were below 0.5 and declined gradually after that for HOTEPk and TEPk; for D1TE purity declined more rapidly. Turning now to coincidence index measures, we see in figure 2B that the two measures based on TE (HOTECI, Delayed TECI) always attain higher true positive rates than the two measures based on cross correlation (NCCCI, NCCHCI). We note that it is not possible to use a coincidence index measure on D1TE, as there is no curve produced by only one delay. The inset of figure 2 B shows that purity again is near 1 for values of TPR below 0.6 and then steadily declines. Of the coincidence index measures, the performances at FPR = 0.01, as attained by HOTECI, TECI, and NCCCI, are statistically equivalent, again also shown in Table 1. The main finding indicated by these results is that, in this network model, extending TE by including multiple delays (delayed TE) greatly improves performance, approximately doubling the TPR of D1TE. Another way of evaluating the performance of these measures was to quantify the total synaptic weight in the network that was associated with correctly identified connections at a relatively low false positive rate. We again selected FPR = 0.01 for this evaluation. At this level, the synaptic weights associated with all correctly identified connections were summed, and then divided by the total synaptic weights in the network. The resulting fractions for each measure are given in column two of Table 1. Note that for all measures used, the fraction of synaptic weights correctly identified was greater than the true positive rate. This indicated that these methods on average identified stronger synapses more easily than weaker ones. As might be expected, weaker synapses were more difficult to identify, but these synapses were also less consequential. The best performance was given by HOTECI, where at FPR = 0.01 the fraction of the total synaptic weight that was associated with true positive connections was 0.851±0.060. For HOTEPk, at FPR = 0.01, the fraction of the total synaptic weight that was associated with true positive connections was 0.791±0.102. The worst performance was given by conventional D1TE, which identified 0.457±0.133 of the available synaptic weight correctly. Note that this is significantly less than the fraction identified by extended versions of TE.

**Table 1:** Fraction of synaptic weights and TPR at FPR = 0.01.

Measure	Fraction synaptic weights	TPR
HOTEPk	0.791±0.102	0.662±0.130
HOTECI	0.851±0.060	0.734±0.084
TEPk	0.750±0.090	0.608±0.108
TECI	0.821±0.055	0.692±0.076
NCCPk	0.763±0.049	0.606±0.062
NCCCI	0.791±0.050	0.649±0.064
D1TE	0.457±0.133	0.355±0.103

The normalized peak cross-correlation histogram (NCCHPk), the normalized coincidence index cross-correlation histogram (NCCHCI), and D1TE had considerably inferior performance compared to the other measures. Because of this poor performance, these three measures were excluded from further investigation.

**Figure 3:**
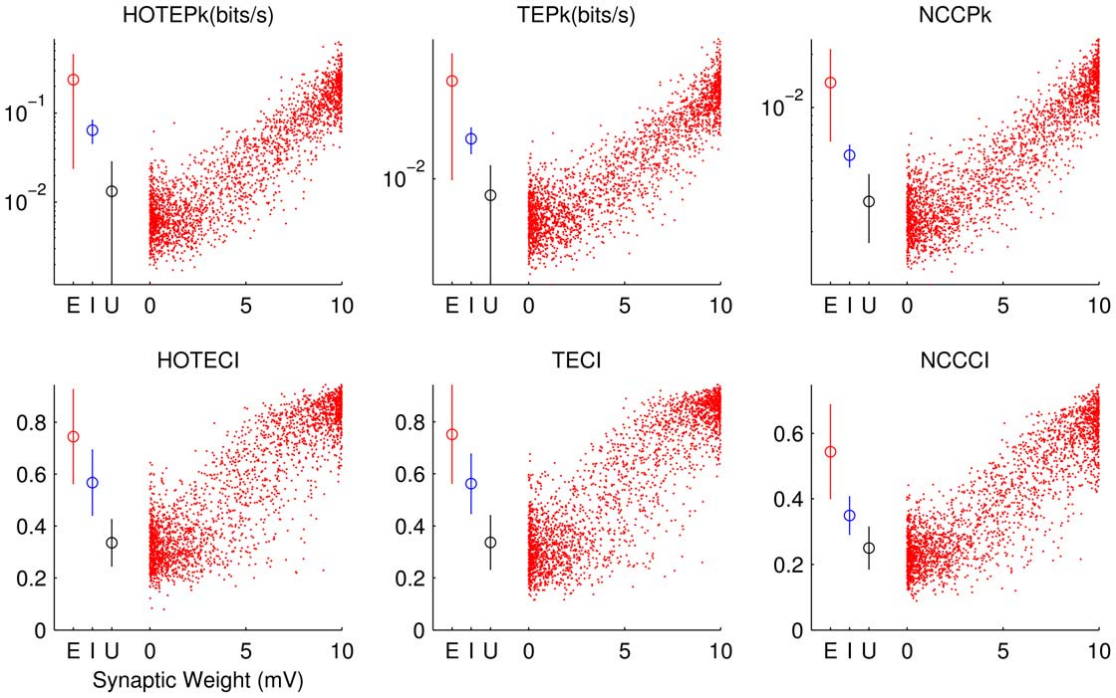
Relationship between effective connectivity measures and synaptic weights. Scatter plot of the values from each connectivity measure (e.g. HOTEPk) against synaptic weights from the model for all 8 model simulations. The synapses which have exactly 10 mV are omitted for clarity. On the left of each plot, mean and standard deviation of all the excitatory connections (E; including 10 mV), inhibitory connections (I), and unconnected pairs (U) are shown. Bars show standard deviations. Peak values (top row) are plotted in log scale, while coincidence index values (bottom row) are plotted in linear scale. One can see that each measure scales with synaptic weight, but that only strong (∼5 mV) synaptic weights are above the values from unconnected pairs.

We further investigated the relationship between synaptic weights from the model and connectivity measures inferred from spike train data (HOTEPk, TEPk, NCCPk, HOTECI, TECI, NCCCI). Figure 3 shows that all these measures generally increased with synaptic weight, but that there was substantial variability. In particular, it is clear that synaptic weights in the model were bimodally distributed, clustering around 0 mV and 10 mV. Intermediate synaptic weights, near 5 mV, often produced connectivity measures that were approximately equal to those produced by unconnected synapses (0 mV). Recall that all inhibitory synapses in the model had a weight of 5 mV, whereas excitatory synapses could vary between 0 and 10 mV. To examine how these different synaptic types could be distinguished, we plotted the average connectivity measure for all excitatory synapses (red circle on left of each plot; lines show standard deviation) along with the average connectivity measure for all inhibitory synapses (blue circle on left of each plot) and unconnected synapses (black circle). As expected, excitatory synapses had the largest average connectivity values, followed by inhibitory synapses and then unconnected synapses. Note that for connectivity measures that used the coincidence index, excitatory synapses and unconnected synapses did not have overlapping standard deviations. In contrast, for connectivity measures that used peak values, inhibitory synapses and unconnected synapses did not have overlapping standard deviations.

**Figure 4:**
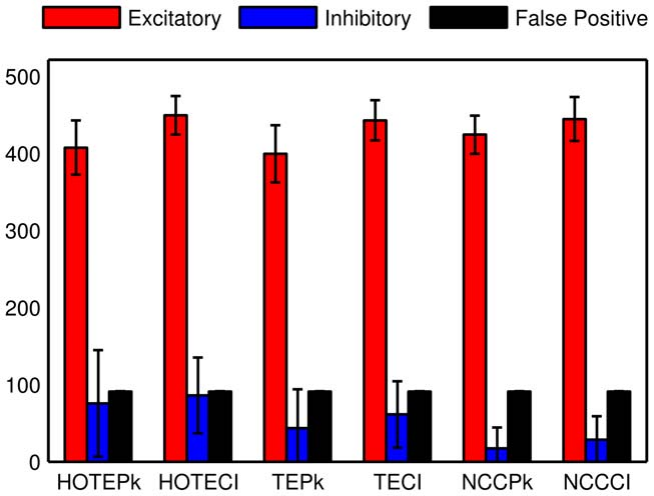
Number of each type of synapse obtained by each connectivity measure. Number of correctly/incorrectly identified connections for selected measures is counted at FPR = 0.01 categorized into type of the connections. The error is standard deviation from 8 model runs. False connections make up 15–20% of the identified connections, although FPR = 0.01. Note that black bars are false positives.

We also examined the number of each type of synapse that these measures identified. Figure 4 shows, in histogram form, the number of excitatory (red), inhibitory (blue), and false positive (black) connections identified by each measure. Several features are evident from this figure. First, the total number of correctly identified synapses (excitatory and inhibitory) was at least five times the number of incorrectly identified synapses (false positive) for all measures. Second, the fraction of true connections that were inhibitory that were identified by higher-order TE measures (HOTECI, HOTEPk) were (0.1608, 0.1567), which nearly reflected the proportion of inhibitory neurons in the model (I/(I+E)  = 0.20). This was to be expected, as each neuron, regardless of type, was given an equal number of connections. In this regard, other measures (TECI, TEPk, NCCCI, NCCPk) did not fare as well, producing ratios of (0.1219, 0.0984, 0.0605, 0.0388) respectively. The poor performance of the correlation measures is not surprising, as inhibitory connections produced dips in correlograms, rather than peaks. Although we took this into account by measuring the peak of the absolute value of NCC, the error bars suggest large variability, and the number of inhibitory neurons found by NCC was always less than or equal to the number found by TE and HOTE (in 8 out of 8 model runs) either by peak values or CI. The problem of identifying inhibitory connections was not as severe for TE measures, as they could detect nonlinear relationships between inputs and outputs. Overall, these results indicate that higher-order TE is more accurate than the other measures tried here.

### Longer recordings improve identification of connectivity

**Figure 5:**
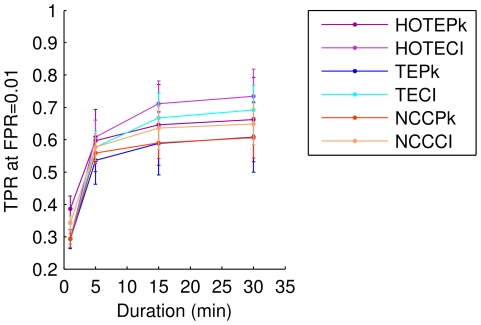
Identification improves with longer recording durations. The true positive ratio (TPR) for each method is plotted against recording duration. All TPRs were measured at a fixed false positive ratio of FPR = 0.01. Error bars indicate standard deviations from 8 model runs.

Another important issue in identifying effective connectivity concerns the quality of conclusions that can be drawn from limited data sets. To explore this issue, the methods were applied to data sets of different lengths. Figure 5 shows the true positive ratio (TPR) measured at FPR = 0.01 for each measure for recordings that were 1 min, 5 min, 15 min and 30 min long. The clear trend is that longer recording times improved the true positive ratio. Interestingly, the increase in performance from 15 min to 30 min was relatively small, suggesting saturation. Somewhat unexpectedly, one could still correctly identify 30% of the connections with 1 min of data. In this respect, NCCCI performed slightly better than TE on 1 min of data. This was perhaps to be expected, due to the finite sampling problem that occurred with TE at very small numbers of spikes.

### Bin size affects performance

**Figure 6:**
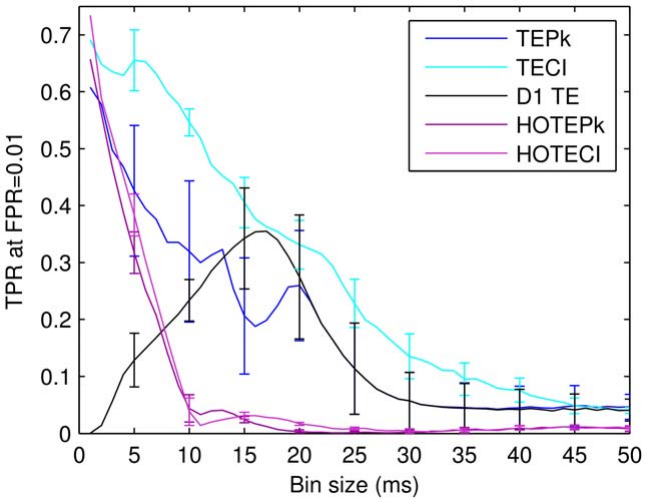
Short bin widths generally improve performance. Comparison of connectivity measures at different bin sizes. The true positive ratio of all measures (taken at FPR = 0.01) is plotted against bin sizes ranging from 1 to 50 ms. The performance of most measures peaks at 1 ms bin size, except for D1TE, which peaks at 17 ms bin size. For HOTEPk, we used order k = 1, l = 3, and for HOTECI, k = 3, l = 2. These parameters were selected because they maximized performance for 1 ms bins. The order was not optimized for larger binning sizes (see text). Beyond 25 ms, performance of all the measures is very low suggesting that one needs high temporal resolution to see effective connectivity between neurons. Error bars indicate standard deviations from 8 simulations, and are shown only every 5 ms for clarity.

As mentioned in the introduction, one way to avoid having to calculate TE at various delays would have been to enlarge the size at which the data were binned. This strategy could conceivably allow synaptic connections with long delays to be correctly identified by D1TE. To investigate this approach, the performance of many measures (TEPk, TECI, D1TE, HOTEPk, HOTECI) was compared and plotted in figure 6. The number of bins used for the peak window of the CI was chosen so that the peak window would be closest to 5 ms, regardless of the bin size (e.g., 2 ms bin → 3 bins, 4 ms bin → 1 bin). Several results are evident from this figure. First, increased bin width indeed improved the performance of D1TE. This measure had its highest TPR (0.35) at a bin width of 17 ms, which is close to 20 ms, the longest synaptic delay built into the model network. Second, the best performance in every measure except D1TE was achieved at the shortest possible bin width of 1 ms. This suggests that high temporal resolution recordings will be best for identifying effective connectivity. Third, the best performance of traditional D1TE (0.36 TPR at 17 ms bin width) was only about half as good as the performance of extended TE measures (HOTECI: 0.73 TPR at 1 ms bin width; HOTEPk: 0.69 at 1 ms bin width). This showed that extensions to TE substantially improved performance. Fourth, all measures performed poorly (TPR≤0.25) at bin sizes greater than 25 ms, again suggesting that high-resolution temporal recordings will be needed to identify effective connectivity in physiological data. Fifth, note that the curves for TEPk and D1TE were nearly identical for bin sizes 20 ms and greater. This was expected because nearly all meaningful information between a neuron pair (in this case, monosynaptic communication) was contained in the first delay.

As a final point, we should note that the higher-order TE measures fell dramatically for bin widths larger than 1 ms. This is probably not a result of much generality, though, as not all parameter values for HOTECI and HOTEPk were explored for this figure. Before being used to generate data for figure 6, different values of *k* and *l* (to vary the order of the presynaptic and postsynaptic message lengths, respectively) for HOTECI and HOTEPk were tried on data binned at 1 ms, and those values that provided the best performance were used in figure 6. If this optimization procedure had been tried each time at every bin width, the curves might not have dropped so precipitously. We did not perform this more general optimization, however, because other results suggested that best performance would be achieved at the smallest bin widths.

**Figure 7:**
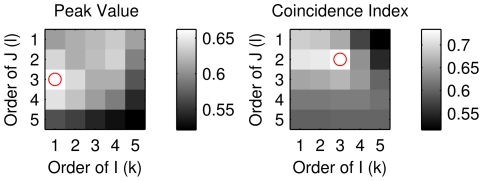
Optimization of higher-order TE (HOTE). With HOTE, the message length of presynaptic neuron *J* ‘s history (*l*) and postsynaptic neuron *I* ‘s history (*k*) could vary from 1 to 5. We measured the true positive ratio, TPR, at a false positive ratio of FPR = 0.01 for all the combinations of *l* and *k* for simulated data binned at 1 ms. The best combination of *l* and *k* is indicated by a red circle. For HOTEPk (left plot), *k* = 1, *l* = 3 was best, and for HOTECI (right plot), *k* = 3, *l* = 2 was best. TPR values are indicated by grayscale bars at the right of each plot.

Figure 7 shows the results of this optimization procedure for HOTEPk and HOTECI. The red circle indicates optimum performance, from where we selected the best values of *k* and *l* for data binned at 1 ms. It is interesting to note that while message lengths longer than 1 bin generally improved performance, the longest message lengths (*k* = *l* = 5) were not the best. Another notable feature of this result is that the optimum order for both HOTEPk and HOTECI is not the same. In fact, the optimal surfaces for these two measures were quite different, the main difference being that HOTEPk was worst at high values of *l*, whereas HOTECI was worst at low values of *l*. Further research will be needed to clarify these differences.

### Computational performance of TE

**Figure 8:**
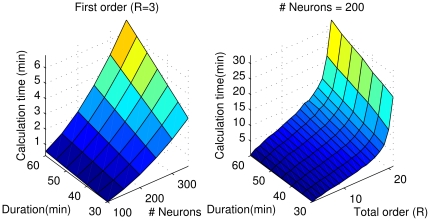
Computational performance of TE algorithms. Algorithms were evaluated on randomly generated Poisson spike trains with mean firing rate of 7 Hz, variable duration, number of neurons, and order. Left panel shows how calculation time scales with the duration of the data set and the number of neurons sampled, for first-order TE. The total order, R, or number of bins used in first order calculations, is 3 (R = 3; one for neuron*I* and two for neuron *J*). Right panel shows how calculation time scales with the duration of the data set and the total order, R, up to R = 20, for 200 neurons sampled. The time required for computation scales linearly with duration and quadratically with the number of neurons (linear with the number of pairs). The time scales almost linearly up to R∼17, and then becomes nearly exponential. All calculations were performed with the freeware offered in this paper, run on a 3.4 GHz Intel Xeon computer with 32 GB RAM, Ubuntu Linux 11.04 32-bit, running the freeware version of Matlab, Octave 3.2.4. No parallelization was used for this performance analysis. Note that for our optimal order R = 5 or 6, one hour data sets of 200 neurons can be analyzed at all delays (1–30 bins) in ∼5 min, making this algorithm potentially useful for large physiological data sets.

As experimental data sets increasingly contain time series from 100 or more spiking neurons [12], [74], the computational efficiency of algorithms measuring connectivity has become an important issue. To investigate how computation time scaled for the higher-order TE algorithms used here (HOTECI, HOTEPk), we tested the algorithms at various message lengths (3–20) on model data sets with various numbers of neurons (100–300) and recording lengths (30–60 min). The firing rate is fixed at 7 Hz for all the neurons in this test. Figure 8 shows that even for relatively large data sets containing 200 neurons recorded for 1 hr, higher-order TE can still be computed in ∼5 min with standard computational resources available to many labs. For D1TE, computation time, *T*, scales as
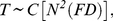
(12)where *C* is a constant that takes into account machine-specific factors and the desired time units, *N* is the number of neurons, *F* is the firing rate, and *D* is the duration of the recording, in number of time bins. For HOTE, the relationship is slightly more complex, and the computation time, *T*, scales as
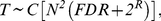
(13)


where *R* is the total order (*k* + *l +* 1) used in the calculations. The derivation of these relationships, as well as other details of the algorithms, can be found in the supporting information (Document S1).

## Discussion

### Main findings

In this work, we have extended single-bin, single-delay transfer entropy to accommodate a range of delays and message lengths. We found that these extensions doubled the rate at which effective connections were correctly identified in a spiking cortical network model. Moreover, 85% of the total synaptic weight was associated with true connections. Fortunately, even for 1 hr recordings of 200 neurons, these calculations could be performed in ∼5 min on a currently typical research computer. We offer the code as freeware, and suggest that it may soon be applicable to physiological data sets.

### Relation to existing work

Although there is a large and growing literature on measuring various types of connectivity in complex neural networks [6]–[8], somewhat fewer papers have been devoted to measuring effective connectivity in networks of spiking neurons [16], [25], [39], [43], [45], [64]. In general, this subset of papers has demonstrated that it is possible to correctly identify effective connections between spiking neurons in many cases. For example, Cadotte and colleagues showed that Granger causality could be used to find a chain of connected neurons with elevated synaptic strengths that was embedded in a realistic spiking network model [25]. This is a particularly difficult task when there are many configurations of synaptic connections that can lead to false positive identifications. Further, Cadotte et al demonstrated that conditional Granger causality could be used to eliminate many of these spurious connections [25]. With these methods, they also estimated changes in synaptic strengths in data from dissociated cultures grown on 60 channel microelectrode arrays [25]. In other work, Garofalo and colleagues applied several different measures, including D1TE, to a pulse-coupled cortical network model [16]. They found that TE was the best of these methods and went on to apply it to data from dissociated cultures grown on 60 channel microelectrode arrays, obtaining effective connectivity maps [16].

We have built upon these important results by extending TE to have a range of delays and message lengths. These extensions have improved the true positive rate from 0.36 for D1TE to 0.73 for HOTECI, a 100% increase, on the cortical network model used here. This improvement was expected, as signals between neurons in this spiking cortical network model had a wide range of delays. This dramatic improvement in accuracy would not have been possible, however, without the development of rapid algorithms. Now, higher order TE can be evaluated at over 30 different delays for all neuron pairs in a 200 neuron network in about 5 minutes, something that was previously impractical.

We should note that the full benefits of higher order TE were probably not utilized in this spiking cortical network model, as intrinsically bursting neurons were not included. We expect that higher order TE will show itself to have significantly superior performance to delayed TE only in this context. Including intrinsically bursting neurons in the network model to test this possibility will be an important topic of future research.

Previous work by others has stressed the importance of using conditional measures to eliminate spurious connections [25]. In such measures, the connectivity between two neurons is not determined by a pairwise measurement alone, but on information about surrounding neurons as well. Indeed, conditional measures have been shown to improve accuracy in identifying connections [25], [75]. In the present work, we did not adopt this approach and instead used only pairwise measures. We showed that pairwise measures at multiple delays could, by themselves, provide substantial knowledge about connectivity; we consider this an important step in a program to apply TE to complex neural networks. We have used conditional TE measures and found that they improve prediction still more (unpublished data), and we plan to address this complex topic fully in a future paper.

In the literature, delayed peaks in cross-correlograms have often been used to infer connections between neurons [53]. Here we showed that in general NCC performed almost as well as HOTE, and that NCC was clearly better for short recording durations. However, HOTE was superior in identifying connections from inhibitory neurons, and the performance of HOTE surpassed that of NCC in most respects for recording durations of 30 min or more.

### Validity of results

The results presented here constitute a proof of principle that extended versions of TE can be successfully applied to spiking cortical network models. It remains to be seen whether or not this method can produce similar results when it is extended to data sets from living neural networks. Two important issues are relevant to this extension.

First, is the model that we have used realistic enough? Despite the many realistic features noted in the introduction, in several respects this model is different from living neural networks. For example, the firing rate of the model neurons was nearly fixed for each type of neuron. In real networks, firing rates are reported to follow a log-normal distribution [76]. In addition, the synaptic weight distribution in the model was bimodal, with nearly all synapses having either maximal or minimal strengths. This disagrees with data from living neural networks, where synaptic weights are reported to follow a log-normal distribution [77]. The topology of connections in the model network was random, but most current reports suggest that living cortical networks follow a scale-free or small-world topology [78]–[82]. The number of synapses made by a cortical neuron is also known to fall off with distance [83], [84], something that was not included in this model. Another relevant issue is the degree to which we sub-sampled the broader network. We successfully determined much of the connectivity among 100 neurons sub-sampled from a 1,000 neuron network (a 10% subsample), suggesting that problems introduced by sub-sampling may be tractable. However, in physiological experiments the degree of sub-sampling is probably much worse than in our simulations. For example, there may be as many as 60,000 neurons over our recording array, of which we may be able to record 300 (a 0.5% subsample). Although many of the neurons in cortex fire very rarely and may not contribute much to total spike activity [85], this issue of sub-sampling needs to be explored further; we plan to do so in future work. Perhaps equally important, the model network that we used did not produce network bursts, also known as synchrony, which are known to be common in living hippocampal and cortical networks [74], [86]. In network bursts, many neurons fire in a relatively short period, typically in the range of 100 ms [87]–[89]. Such bursts produce elevated correlations between all neuron pairs in a network, leading to problems in identifying connections through TE and CC. In such a circumstance, structurally unconnected pairs can have significant TE or CC values, and effective connectivity will no longer be a subset of structural connectivity. Our study confirmed that the method identified structural connectivity of the model network as effective connectivity in stationary data. This problem of network bursts should be investigated further before HOTE and CC are applied to systems that have large network bursts. To the extent that the model used here is unrealistic, HOTE may not be as successful when applied to recordings from live neurons.

Second, in actual data where physical connectivity is not known beforehand, it will be necessary to distinguish true from spurious connections based on HOTE values alone. How can a threshold be selected to accurately separate these? Without knowing physical connectivity in a living neural network, it will not be possible to set a threshold at a false positive rate of 0.01, for example. Null models of the data produced by shuffling may provide some help in establishing a threshold [90]–[93]. This is clearly a difficult problem and beyond the scope of the present paper. However, this issue must be solved if these methods are to be applied to experimental data sets successfully.

In conclusion, extended versions of TE still face issues related to sub-sampling, bursting activity, and determining a suitable threshold in physiological data. If the errors from these issues can be eliminated or sufficiently bounded, then higher-order TE may become a useful tool for determining effective connectivity for experimentalists recording from hundreds of spiking neurons.

## Supporting Information

Text S1 **Transfer entropy toolbox project website address.** http://code.google.com/p/transfer-entropy-toolbox/
(TEX)

Code S1 **Izhikevich's program for cortical network simulation.** Copied from [58].
(M) Click here for additional data file.

Document S1 **PDF document of algorithm description of transfer entropy calculation.**
(PDF)
