# Supplementary material for: Extending Transfer Entropy Improves Identification of Effective Connectivity in a Spiking Cortical Network Model
Source: PLoS One. 2011 Nov 15;6(11):e27431. doi: 10.1371/journal.pone.0027431 (PMC3216957; doi:10.1371/journal.pone.0027431)
Supplement: Document S1 — PDF document of algorithm description of transfer entropy calculation. (PDF) [file pone.0027431.s003.pdf]

First-Order Algorithm

Transfer entropy between two neurons i and j is computed in two main steps:

1. For all time bins t, count the number of distinct firing patterns at i_{t+1}, i_t, and j_t. This is done to estimate transition probabilities (e.g. $p(i_{t+1}, i_t, j_t)$). For first-order TE, there are $2^3 = 8$ possible patterns because a neuron is either spiking or not in a given time bin.

2. Using the estimated transition probabilities, compute the TE from $j \Rightarrow i$ (T_{ij}) by using Equation 1 below.

$$T_{ij} = \sum_t p(i_{t+1}, i_t, j_t) \log \frac{p(i_{t+1}|i_t, j_t)}{p(i_{t+1}|i_t)} \tag{1}$$

The first step is computationally expensive, so care must be taken to minimize the number of steps taken for each pair of neurons. One way to picture the algorithm is shown in Figure 1, where a copy of the time-series for neuron i is shifted backwards by one time bin (neuron i') and stacked on top of the time-series for neurons i and j. This copying and shifting of i allows us to look at each column of the stacked series and simply count the distinct patterns observed[1]. If we visit every column, we will have counted up all firing patterns present in the data and can proceed to estimate the transition probabilities.

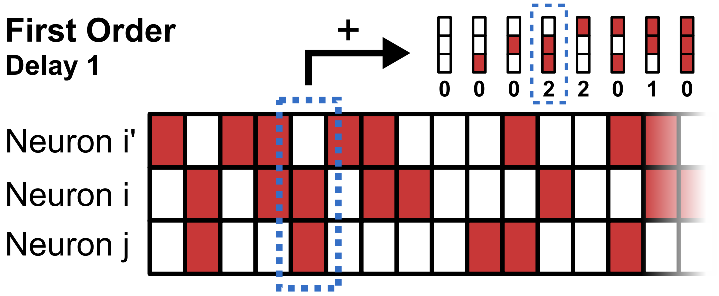

Figure 1: *First-order transfer entropy with a delay of one time bin. The time-series for neuron i' is a copy of the time-series for neuron i, but shifted back by one time bin.*

At a high-level, the first-order algorithm looks something like Listing 1. For each pair of N neurons, we count up the firing patterns for all time bins $t \in \{1 \ldots D\}$, where D is the duration of our data, and then use those counts to compute the TE from $j \Rightarrow i$. While this algorithm works fine for dense time-series with a large proportion of spikes to time bins, it is unnecessarily slow for sparse time series. When the proportion of spikes to time bins is

[1] In practice, we do not have to physically copy the time-series for neuron i. Instead, we maintain an additional pointer into the time-series, shifted appropriately.

low, the majority of firing patterns will be empty (i.e. no neurons are spiking). How can we use this to our advantage?

Listing 1: *First-order transfer entropy algorithm for dense time-series*

```
% N is the number of neurons
% D is the total number of time bins (duration)

for i = 1:N
  for j = 1:N
    ij_series = [shift(time_series(i), -1), time_series(i), time_series(j)]

    % Count patterns for all time bins
    counts = zeros(1, 2^3)
    for t = 1:D
      pattern = ij_series(:, t) % Get firing pattern at time t

      % Count patterns (0-7)
      counts(pattern + 1) = counts(pattern + 1) + 1
    end

    % Calculate TE from j -> i
    for c = counts
      ... % Estimate transition probabilities
    end
  end % for j
end % for i
```

Because most time bins will have no neurons spiking, we only need to visit the time bins where at least one of our series (i', i, and j) has a spike. The number unvisited time bins will then be the count of our "no spike" firing pattern. If we assume that our time-series are stored as a series of integers representing the time bins that a given neurons was spiking, the algorithm will look like Listing 2.

We use the `current` and `move_next` functions to represent a pointer into each time series that can be moved forward. Instead of visiting every time bin, t now jumps around to just the non-empty bins by moving to the minimum `current` value of all time-series. For each visited bin, we count of the firing patterns as before, but we need to compute the number of unvisited time bins (empty firing pattern) before calculating the TE from $j \Rightarrow i$.

Listing 2: *First-order transfer entropy algorithm for sparse time-series*

```
% N is the number of neurons
% D is the total number of time bins (duration)

for i = 1:N
  for j = 1:N
    ij_series = [shift(time_series(i), -1), time_series(i), time_series(j)]

    % Start off at the first time bin with a spike (i', i, j)
    t = min(current(ij_series(1)),
```

```
            current(ij_series(2)),
            current(ij_series(3)))

    % Count patterns for all time bins with spikes
    counts = zeros(1, 2^3)
    visited = 0

    while t <= D
      pattern = 0

      % Go through i', i, and j series
      for series_idx = 1:3
        % Check for a spike at time t
        if current(ij_series(series_idx)) == t
          % Update firing pattern
          pattern = bitset(pattern, series_idx)

          % Move the sparse time series forward
          move_next(ij_series(series_idx))
        end
      end % for series_idx

      % Get the next closest time bin with a spike
      t = min(current(ij_series(1)),
              current(ij_series(2)),
              current(ij_series(3)))

      % Count patterns [0, 8)
      counts(pattern + 1) = counts(pattern + 1) + 1
      visited = visited + 1
    end

    % All unvisited time bins have no spikes (pattern 1)
    counts(1) = D - visited

    % Calculate TE from j -> i
    for c = counts
      ... % Estimate transition probabilities
    end
  end % for j
end % for i
```

For delayed TE, we would like to calculate the TE between i and j for multiple delays. This is easily accomplished with the algorithm above by shifting the time-series for neuron i (and also for i') backwards by some delay d.

First-Order Algorithmic Complexity

In order to predict the calculation time for a given data set, we need to identify which aspects of the TE algorithm significantly affect performance. We can then derive a formula for estimating the amount of time it will take to run any TE calculation.

The number of neurons N will definitely affect performance, since this quantity is used in the two outer `for` loops in Listing 2 on lines 4 and 5. In fact, if we assume the body of the j loop (starting on line 6) will take c seconds each time to run, then calculating TE for one delay time will take roughly cN^2 seconds. In computer science terminology, this means the calculation time will grow *quadratically* with the number of neurons.

What other factors will affect performance? The number of time bins we have to visit will certainly have an impact. For dense time-series, this will be D time bins. Sparse time-series, however, we require that we go through and count how many bins were visited for all pairs of neurons. Unfortunately, doing this is almost as computationally expensive as calculating TE in the first place! If we know the average firing rate of our neurons F, though, we can estimate the number of visited time bins for any pair of neurons by calculating FD. When our neurons all have a fairly consistent firing rate, this estimate will work well. As D increases, then, we should expect the calculation time to (roughly) grow *linearly* due to FD being linear in D.

For first-order TE, estimating the transition probabilities (line 46 of Listing 2) for a pair of neurons does not take any longer for different data sizes. We can therefore bundle this and other machine-specific details into a constant C, making our final calculation time formula:

$$C[N^2(FD)] \tag{2}$$

where N is the number of neurons, D is the duration (number of time bins), F is the average firing rate, and C is a constant that depends on the actual machine and desired time units.

Higher-Order Algorithm

Our higher-order TE algorithm is a straightforward generalization of the first-order algorithm in Listing 2. We consider more time bins in either the receiving or sending neuron (or both) by stacking additional, shifted copies of their respective time-series (see Figure 2). As before, we then count up the distinct firing patterns by looking at the columns of our stacked series. Estimating the transition probabilities now requires that we take the additional time bins into account, resulting in Equation 3.

$$T_{ij} = \sum_t p(i_{t+1}, i_t^{(k)}, j_t^{(l)}) \log \frac{p(i_{t+1}|i_t^{(k)}, j_t^{(l)})}{p(i_{t+1}|i_t^{(k)})} \tag{3}$$

where k is the order of the receiving neuron i and l is the order of the sending neuron j.

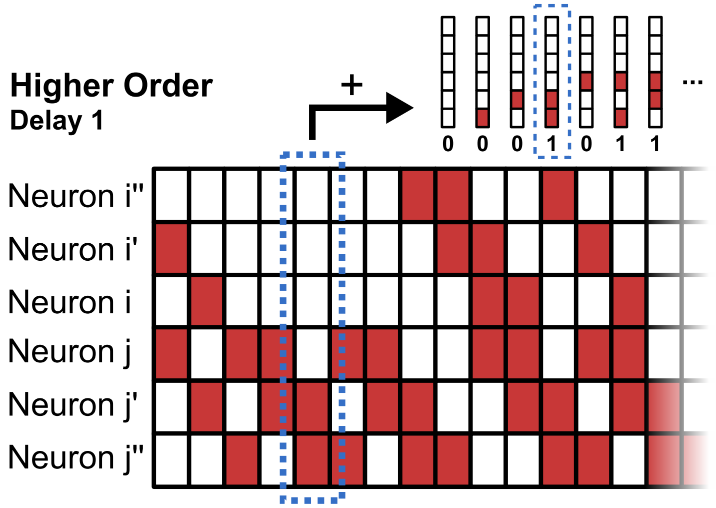

Figure 2: *Higher-order transfer entropy with a delay of one time bin. The time-series for neurons i', i'', j', and j'' are shifted copies of their respective neuron's time series (backward for i, forward for j).*

The pseudo-code in Listing 3 gives a bird's eye view of the higher-order algorithm. The quantity R represents the total order of the calculation, which is $k + l + 1$. For first-order TE, $R = 3$ and we only had three time-series to deal with. For higher-order TE, `ij_series` will be of length R and the number of distinct firing patterns will be 2^R.

Listing 3: *Higher-order transfer entropy algorithm for sparse time-series*

```
% N is the number of neurons
% D is the total number of time bins (duration)
% R is the total order (k + l + 1)

for i = 1:N
  for j = 1:N
    ij_series = [shift(time_series(i), -1),
                 time_series(i),
                 ..., % k shifted copies of time_series(i)
                 time_series(j),
                 ...] % l shifted copies of time_series(j)

    % Start off at the first time bin with a spike (i', i, j)
    t = min(current(ij_series(1:R)))

    % Count patterns for all time bins with spikes
    counts = zeros(1, 2^R)
    visited = 0

    while t <= D
      pattern = 0
```

```
22
23       % Go through all series
24       for series_idx = 1:R
25         % Check for a spike at time t
26         if current(ij_series(series_idx)) == t
27           % Update firing pattern
28           pattern = bitset(pattern, series_idx)
29
30           % Move the sparse time series forward
31           move_next(ij_series(series_idx))
32         end
33       end % for series_idx
34
35       % Get the next closest time bin with a spike
36       t = min(current(ij_series(1:R)))
37
38       % Count patterns [0, 2^R)
39       counts(pattern + 1) = counts(pattern + 1) + 1
40       visited = visited + 1
41     end
42
43     % All unvisited time bins have no spikes (pattern 1)
44     counts(1) = D - visited
45
46     % Calculate TE from j -> i
47     for c = counts
48       ... % Estimate transition probabilities
49     end
50   end % for j
51 end % for i
```

Higher-Order Algorithmic Complexity

In contrast to the first-order algorithm, higher-order TE depends heavily on the total order R. There are two places in Listing 3 that depend on R in a way that significantly impacts performance. First, the loop starting on line 24 will be run R times for each visited time bin. Remembering that we estimated the number of visited time bins as the average firing rate times the duration (FD), we can estimate the run time of this loop as FDR. Thus, we can at least expect the calculation to grow linearly with R when D and N are held constant.

Estimating the transition probabilities no longer takes a fixed amount of time. Instead, starting on line 47, it will take some amount of time dependent on the length of the `counts` variable. As there are 2^R distinct firing patterns, `count` will have size 2^R. This means, unfortunately, that the calculation time will grow *exponentially* with the total order. In other words, we expect the calculation time to roughly double each time we increase R by 1. In practice, however, this behavior does not become an issue until R grows beyond 20 or

so. This is because the actual run time of one iteration of the loop on line 47 is so small doubling it a handful of times makes little difference until R gets large.

Putting it all together, we get the final TE calculation time formula in Equation 4. FDR and 2^R are added together because the 2^R loop is run after the FDR loop (instead of inside it). C is still a constant that represents machine-specific factors and the desired time units.

$$C[N^2(FDR + 2^R)] \tag{4}$$
